# Assessment of Knowledge, Attitudes, and Perceptions Regarding Artificial Intelligence Among Undergraduate Medical Students

**DOI:** 10.7759/cureus.116988

**Published:** 2026-09-27

**Authors:** Sajal Gulati, Chandrakant Kokatanur, Supriya Patil

**Affiliations:** 1 Department of Medicine, Krishna Institute of Medical Sciences, Krishna Vishwa Vidyapeeth (Deemed to be University), Karad, IND; 2 Department of Forensic Medicine and Toxicology, Krishna Institute of Medical Sciences, Krishna Vishwa Vidyapeeth (Deemed to be University), Karad, IND; 3 Department of Community Medicine, Krishna Institute of Medical Sciences, Krishna Vishwa Vidyapeeth (Deemed to be University), Karad, IND

**Keywords:** ai digital health platform, ai in healthcare, artificial intelligence, curriculum, digital health, machine learning, medical education, medical students, predictive analytics and ai, undergraduate medical education

## Abstract

Background: Artificial intelligence (AI) is rapidly reshaping healthcare by improving diagnostic accuracy, clinical decision-making, and patient care. As AI is progressively incorporated into routine clinical care, future physicians must develop the knowledge and competencies required to use these technologies effectively and ethically. However, evidence regarding undergraduate medical students’ readiness for AI-enabled healthcare remains limited, particularly in the Indian context.

Objective: This study aimed to assess undergraduate medical students’ knowledge, attitudes, and perceptions toward the incorporation of AI in healthcare practice and medical education.

Methods: A descriptive cross-sectional study involving 225 undergraduate medical students was carried out at Krishna Institute of Medical Sciences, Krishna Vishwa Vidyapeeth (Deemed to be University), a medical college in Maharashtra, India. A predesigned questionnaire assessed participants’ knowledge of AI, sources of learning, attitudes toward AI in healthcare, perceived impact on future medical practice, and opinions on the inclusion of AI within the undergraduate medical curriculum. The collected data were analysed using descriptive statistical methods, and associations between categorical variables were assessed using the Pearson chi-square test.

Results: Among the participants, 60.0% (135) reported acquiring knowledge about AI primarily through media, while formal training in AI, machine learning, or deep learning was limited. The majority of participants (79.6%; 179) perceived AI as a supportive tool that would complement the role of physicians rather than replace them. However, 41.8% (94) expressed concerns regarding the potential impact of AI on future employment opportunities. Radiology and surgery were identified as the specialties perceived to incorporate AI most extensively. Overall, 60.0% (135) supported incorporating AI into medical education, while 75.55% (170) favored initiating AI competency training during medical school.

Conclusion: Undergraduate medical students exhibited favorable attitudes and perceptions toward AI in healthcare, despite limited formal exposure to AI-related education and training. The findings support consideration of structured AI education within undergraduate medical curricula, while the self-reported nature of the knowledge assessment should be considered when interpreting the findings. Further multicenter studies using validated assessment tools are needed to evaluate AI knowledge, attitudes, and competencies.

## Introduction

Artificial intelligence (AI) has become a transformative technology in healthcare, promising to revolutionize diagnostics, treatment, and patient care. As we stand at the threshold of a new era in medicine, it becomes imperative to gauge the readiness and receptivity of future healthcare professionals to integrate AI into their practice.

In the public health sector, the requirement for skilled human-machine interaction to aid clinical decisions will increase as medicine and healthcare delivery progress into the AI era [1]. Medical students must be well-versed in AI and its benefits in order to enhance healthcare quality, improve accessibility, and reduce costs. Students must also be informed about AI’s flaws, such as prejudice, transparency, and liability. Numerous experts have examined the prospective contributions of AI within healthcare, emphasizing its capacity to enhance medical decision-making and optimize operational effectiveness [2].

The knowledge and attitudes of these students are vital, as they are poised to be at the forefront of the evolving healthcare landscape, wherein AI technologies are becoming increasingly integrated. As current medical students conclude their training, it is foreseen that AI tools in medicine will have become a routine component of clinical decision-making [3].

Exposure to AI among undergraduate medical students remains limited, which can influence their career choices. Hence, assessing the attitudes and existing knowledge of healthcare students is essential, as this can be a valuable approach to identify areas in AI education that require attention from curriculum planners.

In the evolving landscape of modern medicine, where the roles of healthcare providers are being redefined, the integration of AI depends on coordinated efforts among healthcare stakeholders, including physicians and various professionals. It is imperative to gather insights from a diverse group of healthcare students. As AI’s significance in healthcare continues to grow, there is a clear demand for enhanced knowledge and specialized AI training for both physicians and medical students.

With this background, the present study was undertaken among undergraduate medical students at Krishna Institute of Medical Sciences, Krishna Vishwa Vidyapeeth (Deemed to be University), Karad, Maharashtra, India, to evaluate the knowledge and attitudes of medical students toward AI in healthcare. The present study aimed to assess the knowledge, attitudes, and perceptions of undergraduate medical students regarding the role of AI in healthcare and medical education. In addition, the study explored the primary sources of students’ knowledge regarding AI to identify areas that may inform future AI education strategies.

## Materials and methods

Study design

A descriptive cross-sectional study was conducted among undergraduate medical students at the Krishna Institute of Medical Sciences, Krishna Vishwa Vidyapeeth (Deemed to be University), Karad, Maharashtra, India.

The required sample size was determined based on the findings of a similar study conducted by Mehta N et al. [4], using the following formula:

$$n = \frac{4pq}{l^2}$$

where p represents the proportion of students aware of the application of AI in healthcare (83%), q represents the proportion of students unaware of the application of AI in healthcare (17%), and l represents the allowable error (5%). Based on these parameters, the calculated sample size was 225 participants.

Study participants

A total of 225 undergraduate medical students participated in the study. The Google Form (Google LLC, Mountain View, California, USA) was circulated through the WhatsApp groups of all undergraduate medical student batches, from the first professional year through internship, thereby inviting all eligible students to participate. All undergraduate medical students enrolled from the first professional year through internship were eligible to participate. Participation was voluntary, and informed consent was obtained before data collection. The questionnaire was adapted from a previously published study [4] and was distributed electronically through Google Forms (see Appendix).

The first section collected demographic information, including age, gender, academic year, and sources of knowledge regarding AI. The second section assessed participants’ attitudes and perceptions regarding the role of AI in healthcare. The third section assessed participants’ self-reported understanding of AI-related concepts, including AI, machine learning, neural networks, and deep learning. The fourth section assessed perceptions regarding the incorporation of AI into medical education.

Responses to closed-ended questions were categorized as "Yes," "No," or "Maybe," while an "Others" option was provided for participants to specify additional medical specialties.

Ethical considerations

The study was conducted following approval from the Institutional Ethics Committee of Krishna Institute of Medical Sciences, Krishna Vishwa Vidyapeeth (Deemed to be University), Karad, Maharashtra, India (Approval No. KIMSDU/IEC/06/2022). Informed consent was obtained from the participants before data collection. The questionnaire was anonymous, and no personally identifiable information was collected.

Statistical analysis

Data were processed and analyzed using IBM SPSS Statistics version 20 (IBM Corp., Armonk, NY). Descriptive statistics, including frequencies and percentages, were used to summarize the demographic characteristics and responses related to participants’ self-reported understanding, attitudes, and perceptions. The knowledge-related items were analyzed individually as categorical responses, and no composite knowledge score or classification was calculated. Graphical presentations were used where appropriate.

Associations between categorical variables were assessed using the Pearson chi-square (χ²) test. Statistical significance was considered at a p-value <0.05. For inferential analyses, the relevant test statistic, degrees of freedom, and p-value were reported where applicable.

## Results

Of the 225 participants, 83 (36.9%) identified as male and 142 (63.1%) as female; no participants selected "non-binary/others" or "prefer not to say." Age distribution was as follows: 56 (24.9%) in the 18-19 age group (15 males, 41 females), 88 (39.1%) in the 20-21 group (30 males, 58 females), 59 (26.2%) in the 22-23 group (25 males, 34 females), and 22 (9.8%) in the 24-25 group (13 males, nine females). A chi-square test showed a significant association between age and gender (χ² = 8.171, df = 3, p = 0.043). Regarding the academic year, 62 (27.6%) were in the first professional year, 47 (20.9%) in the second, 39 (17.3%) in the third, 45 (20%) in the fourth, and 32 (14.2%) in their internship.

The majority of participants (135; 60%) cited the media as their primary source of AI-related knowledge, followed by the medical school curriculum (37; 16.5%), university-level courses (34; 15.1%), and medical research projects (18; 8%). Only one (0.4%) participant reported not using any of these sources (Figure 1).

**Figure 1:**
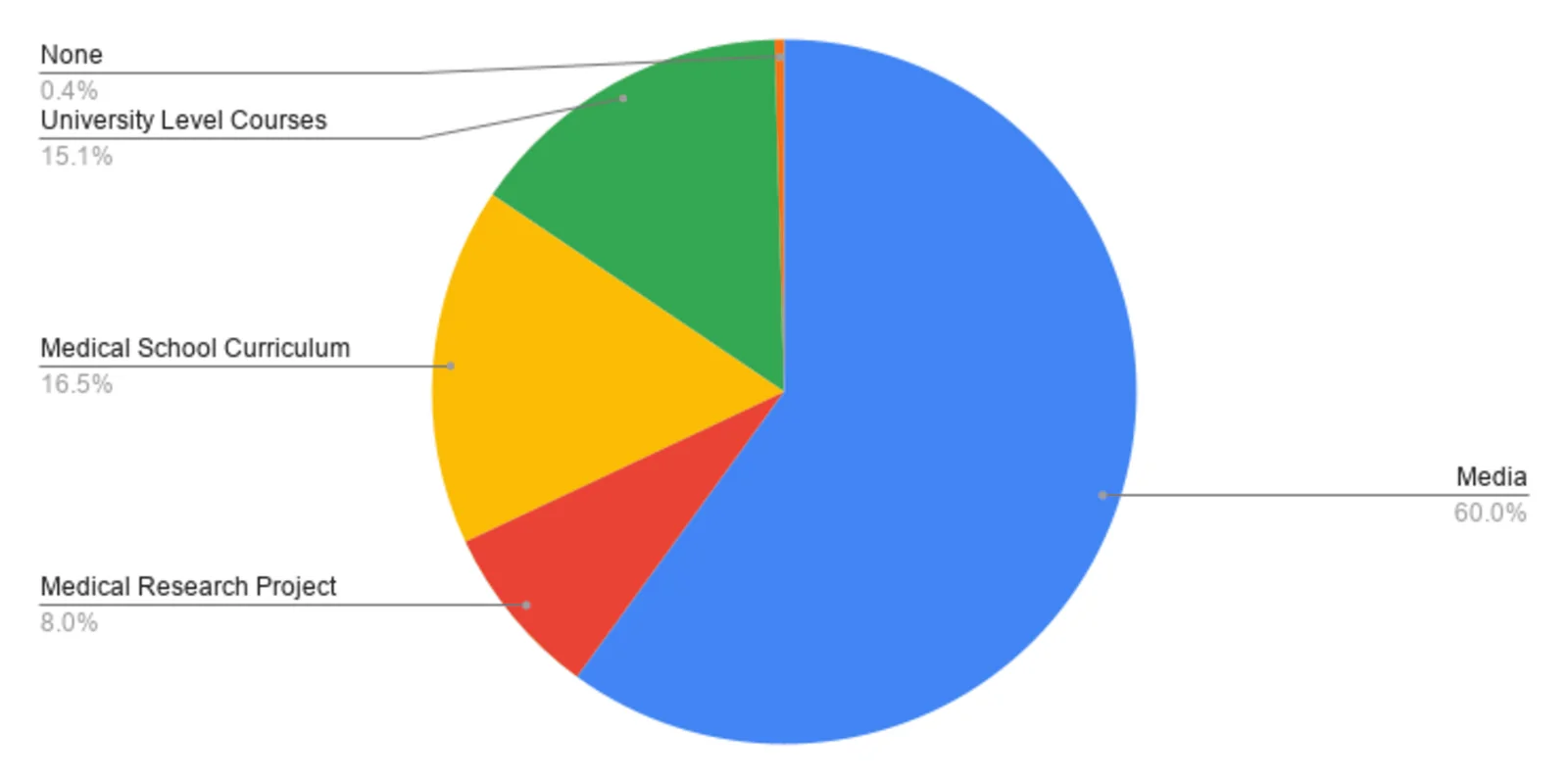
Participants’ sources of knowledge regarding AI

Overall, 61 (27.1%) participants indicated that they had received prior training in computer science, whereas 164 (72.9%) participants had no such training. Regarding AI, ML, or DL courses, 36 (16%) participants had completed one, while 189 (84%) participants had not. In terms of AI-specific lectures, 69 (30.7%) participants had attended, while 156 (69.3%) participants had not. Regarding AI’s role in healthcare, 179 (79.6%) participants viewed AI as a "partner," while 46 (20.4%) participants saw it as a "competitor."

One hundred seventy-four (174; 77.3%) participants anticipated AI in robotic surgeries, 178 (79.1%) in diagnostic imaging, and 188 (83.60%) in decision-making. Fifty-nine (59; 26.20%) participants supported AI in psychiatric counseling, and 103 (45.80%) participants were in favor of empathetic care being provided. In personalized prescriptions, 153 (68%) favored AI, while 72 (32%) did not. Regarding specialty choice, 61 (27.1%) saw AI’s influence, and 137 (60.9%) participants agreed that some medical specialties may be more prone to replacement by AI. Concerns about AI’s impact on jobs were raised by 94 (41.8%), while 153 (68%) viewed AI as crucial in healthcare.

A total of 126 (56%) participants were familiar with "artificial intelligence," 145 (64.4%) with "machine learning," 69 (30.7%) with "neural networks," and 58 (25.8%) with "deep learning." Regarding AI challenges in medicine, 166 (73.8%) acknowledged them. On AI's learning capability, 99 (44%) agreed it can learn from data, and 175 (77.8%) recognized the need for labelled data. In addition, 112 (49.8%) believed AI excels in pattern recognition but struggles with deduction (Figure 2).

**Figure 2:**
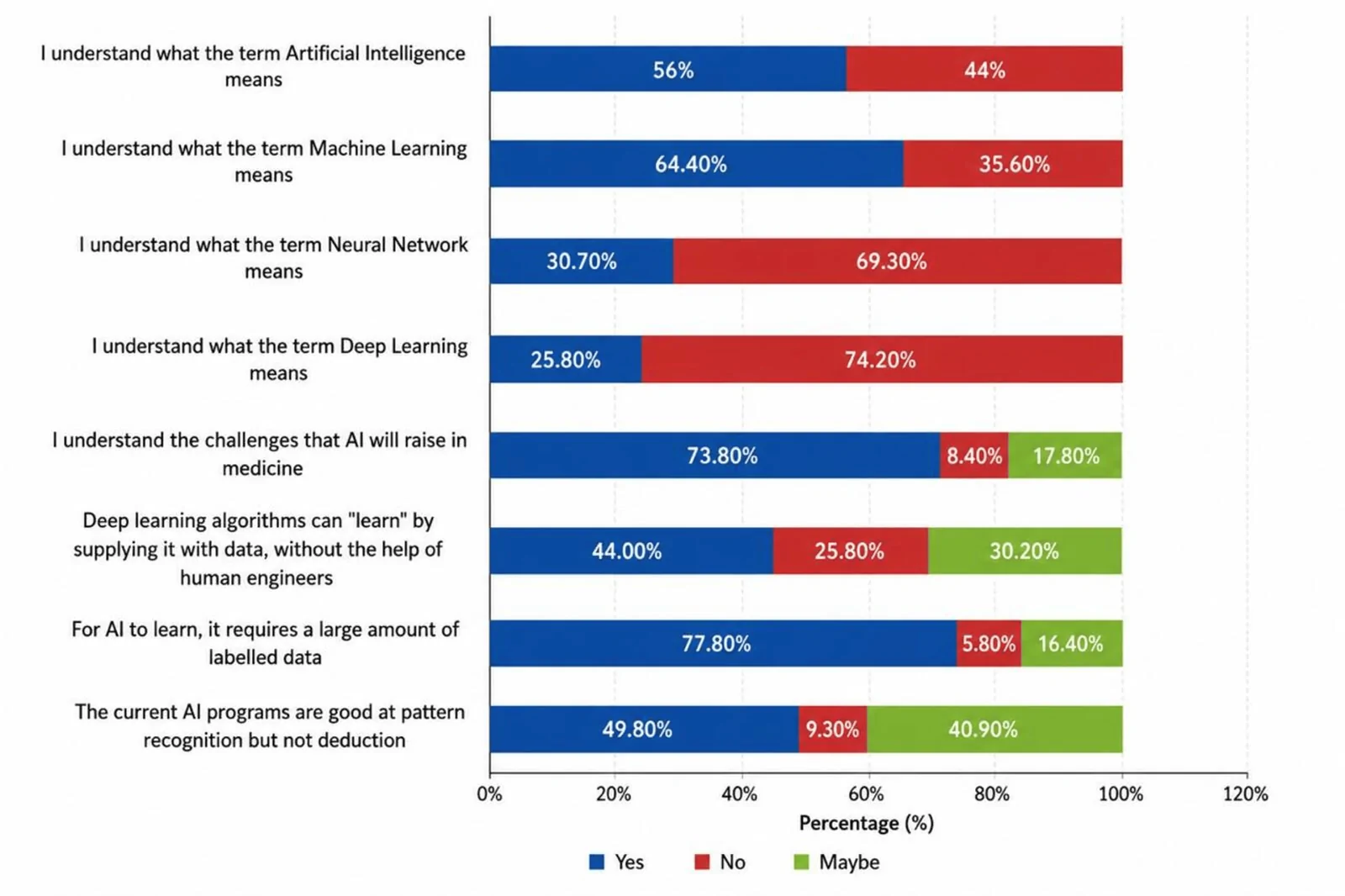
Knowledge and perceptions of study participants regarding AI in healthcare

Radiology was identified as the medical specialty with the highest potential for AI integration by 78 (34.5%) participants. Surgery followed with 63 (28%) participants, and neurology was recognized by 42 (18.5%) participants (Figure 3).

**Figure 3:**
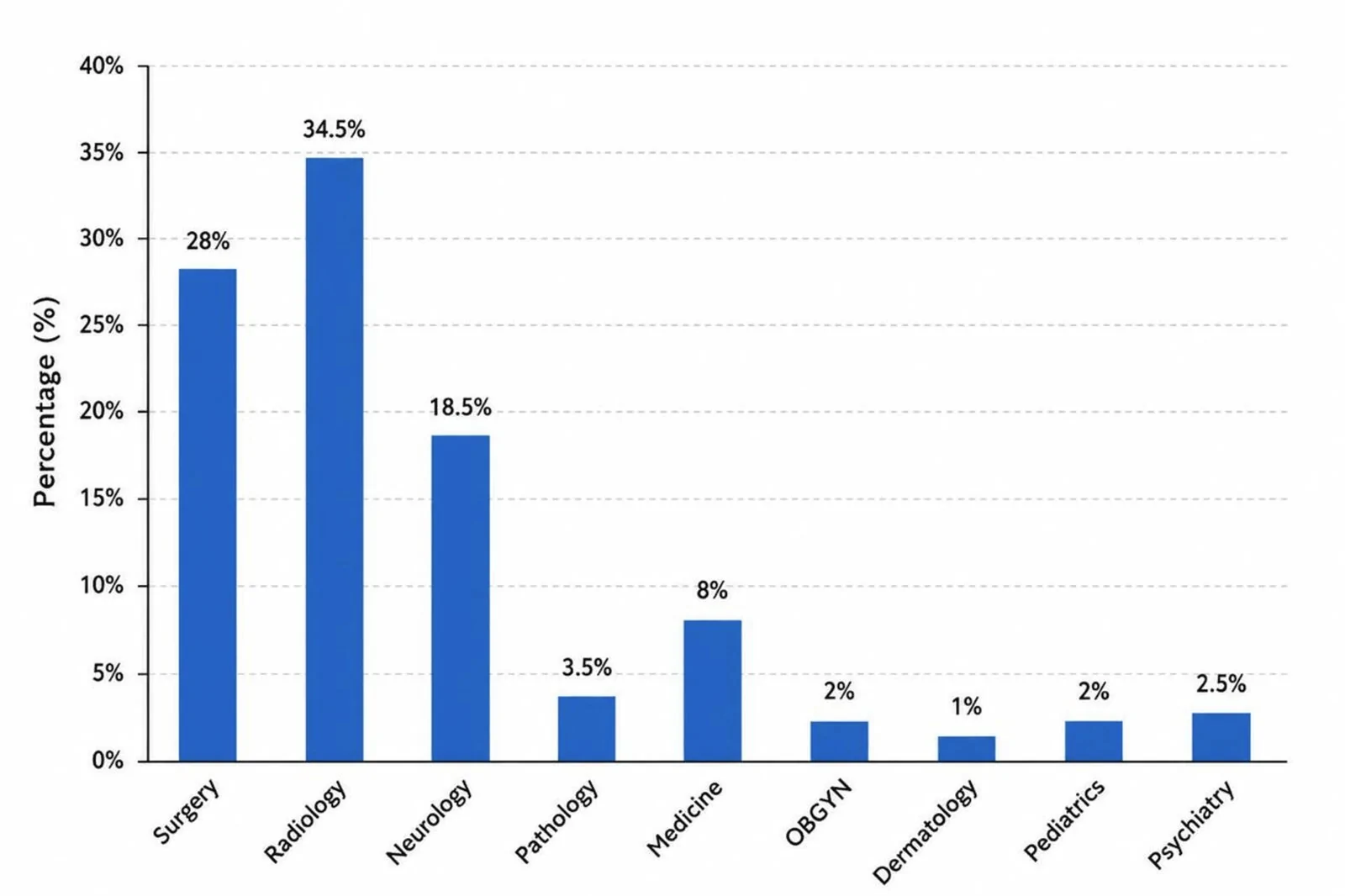
Medical specialty perceived to incorporate AI the most

In the study, 135 (60%) participants supported incorporating AI into medical education, while 35 (15.6%) opposed it, and 55 (24.4%) were uncertain. Regarding AI-generated questions, 76 (33.8%) favored them, 80 (35.6%) preferred traditional questions, and 69 (30.7%) were undecided. On AI-generated assessments with personalized feedback, 80 (35.6%) saw potential, 95 (42.2%) disagreed, and 50 (22.2%) were uncertain. 

Lastly, 91 (40.4%) believed AI-driven clinical exams would offer more objectivity, while 71 (31.6%) disagreed, and 63 (28%) were uncertain.

A majority of participants (170; 75.55%) supported the incorporation of AI competency training during medical school. Thirty-eight (38; 16.89%) participants suggested that it should be introduced during residency, while 11 (4.89%) participants proposed starting the training for practicing physicians. Six (6; 2.67%) participants believed that AI competency training was unnecessary (Table 1).

**Table 1:** Preferred stage to initiate AI competency training

Stage to initiate AI training	N (%)
Medical school	170 (75.55%)
Residency	38 (16.89%)
Practicing physicians	11 (4.89%)
Training not necessary	6 (2.67%)

## Discussion

The sample showed a female predominance, with 142 (63.1%) participants identifying as female and 83 (36.9%) as male, along with a significant relationship between age and gender distribution. These observations contrast with those reported by Ampofo JW et al., who observed a more balanced male-to-female ratio and a greater proportion of participants aged 20-23 years, highlighting regional and institutional differences [5]. Regarding the academic year, 62 (27.6%) participants were in the first professional year, and 32 (14.2%) were in the internship year, aligning with Mehta N et al., and reflecting typical medical student progression [4]. The variation across academic years may influence students’ knowledge and attitudes regarding AI in healthcare.

In our study, the primary source of AI knowledge was the "media" (60%), followed by "medical school curriculum" (16.5%) and "university-level courses" (15.1%), similar to Bisdas S et al., where web browsing (60.1%) and social media (59.4%) were dominant. Interestingly, only 0.4% in our study cited "None" as their source, in contrast to Bisdas S et al., where university courses and friends/family were least relied upon (33.6% and 24.6%, respectively) [6]. The observed differences may be attributed to variations in surveyed populations, access to resources, and personal preferences, highlighting the potential value of educational strategies to improve AI awareness among medical students.

In our study, 27.1% of the participants had prior computer science training; 16% had completed courses in AI, ML, or DL; and 30.7% had attended AI lectures. This contrasts with Mehta N et al., where 39.8% attended AI lectures [4]. These findings suggest limited formal exposure to AI education among medical students, emphasizing the potential value of educational initiatives to develop AI-related knowledge and skills. Incorporating AI into medical curricula and providing hands-on training may help address these educational needs and support students’ preparation for future AI-integrated healthcare.

Our study shows that 68% of participants recognize AI's significant role in transforming healthcare, emphasizing its potential to improve diagnostics, treatment, and administrative processes. This aligns with Bisdas S et al., where 79.6% viewed AI as a partner in healthcare [6]. Both studies highlight the collaborative potential of AI, stressing the importance of a partnership between AI technologies and healthcare professionals.

Our study, compared to Mehta N et al., reveals mixed perceptions among medical students about AI's role in healthcare [4]. While both studies acknowledge AI’s potential in diagnosis and imaging, skepticism remains about AI's ability to provide empathetic care or perform complex procedures like robotic surgeries. This reflects concerns about AI replacing human elements in medicine, such as empathy and personal counseling. Training initiatives should focus on both the technical and ethical dimensions of AI, enabling healthcare professionals to work effectively alongside AI while maintaining compassionate, patient-centered care.

Radiology was identified as the leading specialty for AI integration in our study (34.5%), followed by Surgery (28%). By contrast, Sassis L et al., found Surgery (43.4%) as the dominant specialty, with only 17.3% recognizing Radiology [7]. These differences highlight the evolving nature of AI adoption in healthcare, with Radiology gaining prominence in image processing and diagnostics, while Surgery focuses on procedural technologies. This suggests that AI's role in healthcare is dynamic, influenced by technological advancements and regional or institutional factors.

A significant 73.8% of participants recognized AI’s challenges in medicine, although 17.8% struggled to grasp them, indicating a need for further education. While 44% understood deep learning’s ability to learn from data, 30.2% disagreed, suggesting limited self-reported understanding of this concept. In addition, 77.8% acknowledged the need for large labelled datasets, but 5.8% remained uncertain. In contrast to Doumat G et al., our study found lower support for AI-based clinical assessments and AI-generated learning content [8]. These findings may reflect regional differences in perceptions, underscoring the potential value of focused AI education in medical curricula to address areas of limited self-reported understanding and support students’ preparation for future AI-integrated healthcare.

Our study shows that 60% of participants support including AI in medical training, with 24.4% uncertain and 15.6% opposed. This contrasts with Bisdas S et al., where 85.6% favored AI as a core component of medical curricula. The observed differences could be attributed to regional variations, demographics, and awareness levels. While our study highlights support for AI integration, it also underscores the need for informed discussions to address uncertainties. The stronger support in Bisdas S et al.'s study emphasizes the growing consensus on AI's role in healthcare, calling for medical education to adapt accordingly [6]. 

Regarding the preferred stage for AI competency training, 75.55% (170) selected medical school, emphasizing the need for early exposure to AI concepts. Fewer participants (16.89%) preferred residency training, and only 4.89% supported training for practicing physicians. This aligns with Mehta N et al., where 68% also saw medical school as the ideal time for AI education, reflecting growing recognition of its importance in early medical training [4]. 

Limitations 

This study has some limitations. Its single-center, cross-sectional design limits generalizability and does not allow assessment of changes in perceptions over time. Although the questionnaire was circulated across all undergraduate medical batches, participation was voluntary; therefore, selection and non-response bias cannot be excluded. As the questionnaire was self-administered, responses may be subject to individual interpretation. Additionally, the knowledge-related items assessed self-reported understanding rather than objectively measured AI competency, and separate validation or reliability testing of the adapted questionnaire was not performed. Further multicenter studies using validated assessment tools and larger samples are warranted.

Future directions 

Future research should focus on developing and evaluating structured, competency-based AI curricula that incorporate emerging technologies such as generative AI and large language models into undergraduate medical education. Longitudinal and multicenter studies could assess changes in students’ AI knowledge, attitudes, and competencies following formal training, while also exploring specialty-specific applications. Particular emphasis should be placed on the critical evaluation of AI-generated outputs, recognition of their limitations, and understanding of ethical considerations including privacy, bias, and accountability. Such efforts could help establish standardized AI competencies and prepare future physicians for effective human-AI collaboration while preserving clinical judgment and patient-centered care.

## Conclusions

This study highlights favorable attitudes and perceptions among undergraduate medical students toward the potential role of AI in healthcare, alongside limited formal exposure to AI-related education and training. The findings support the need to consider structured AI education within undergraduate medical curricula, with emphasis on clinical applications, ethical considerations, and interdisciplinary learning. However, the knowledge-related findings reflect students’ self-reported understanding and should not be interpreted as objective measures of AI competency.

Further multicenter and longitudinal research using validated assessment tools is needed to evaluate changes in AI knowledge, attitudes, and competencies and to determine the educational impact of structured AI training.

## Appendices

Questionnaire for the assessment of knowledge, attitudes, and perceptions regarding artificial intelligence among undergraduate medical students

Section 1: Demographics and Prior AI Exposure

1. Age: ____________________ years

2. Gender: a. Male b. Female c. Non-binary/others d. Prefer not to say

3. Academic year: a. 1st Prof year b. 2nd Prof year c. 3rd Prof year d. 4th Prof year e. Internship

4. What is your primary source of knowledge regarding AI? : a. University-level courses b. Medical school curriculum c. Medical research project d. Media e. None

5. Previous training in computer science: a. Yes b. No

6. Completed any course where AI/ML/DL were taught? a. Yes b. No

7. Attended or viewed any talks/lectures on AI? a. Yes b. No

8. Any other training in computer coding/programming? a. Yes b. No

Section 2: Attitudes and Perceptions Toward AI

1. Do you perceive AI in healthcare as a: a. Partner b. Competitor

2. AI will play an important role in healthcare: a. Yes b. No c. Maybe

3. AI will reduce the number of jobs available to physicians: a. Yes b. No c. Maybe

4. Some specialties are more prone to be replaced by AI than others: a. Yes b. No c. Maybe

5. Will AI be able to use patient information to reach a diagnosis? a. Yes b. No

6. Will AI be able to read and interpret diagnostic imaging? a. Yes b. No

7. Will AI be able to create personalized medical prescriptions? a. Yes b. No

8. Will AI do robotic surgeries in the near future? a. Yes b. No

9. Will AI provide empathetic care to patients? a. Yes b. No

10. Can AI provide psychiatric/personal counseling? a. Yes b. No

11. Is the development of AI in medicine affecting your choice of specialty? a. Yes b. No c. Maybe

Section 3: Knowledge of AI

1. I understand what the term Artificial Intelligence means. a. Yes b. No

2. I understand what the term Machine Learning means. a. Yes b. No

3. I understand what the term Neural Network means. a. Yes b. No

4. I understand what the term Deep Learning means. a. Yes b. No

5. I understand the challenges that AI will raise in medicine: a. Yes b. No c. Maybe

6. Which medical specialty do you think incorporates the use of AI the most? a. Surgery b. Radiology c. Neurology d. Pathology e. Internal medicine f. OBGYN g. Dermatology h. Anesthesia i. Family medicine j. Pediatrics k. Psychiatry l. Others: ____________________

7. Deep learning algorithms can “learn” by supplying it with data, without the help of human engineers: a. Yes b. No c. Maybe

8. For AI to “learn”, it requires a large amount of labeled data (information already processed by a human and clearly labeled): a. Yes b. No c. Maybe

9. The current AI programs are good at pattern recognition but not deduction: a. Yes b. No c. Maybe

Section 4: Medical Education

1. Would you feel more objectively assessed and graded if an AI system graded you during the clinical skills exam instead of the usual standardized preceptor? a. Yes b. No c. Maybe

2. If you were to be assessed by an AI system during your clinical skills exam, do you feel that you would receive more personalized feedback than with a human standardized patient? a. Yes b. No c. Maybe

3. Would you prefer learning from personalized AI-generated questions as opposed to traditional questions (from textbooks, question banks, etc.)? a. Yes b. No c. Maybe

4. Should AI competencies be a part of medical training/curriculum? a. Yes b. No c. Maybe

5. What should be the preferred stage to initiate AI competency training? a. Medical school b. Residency c. Practicing physicians d. Training not necessary
